# *Amaranthus hypochondriacus* L. as a Sustainable Source of Nutrients and Bioactive Compounds for Animal Feeding

**DOI:** 10.3390/antiox10060876

**Published:** 2021-05-30

**Authors:** Marianna Oteri, Fabio Gresta, Annalisa Costale, Vittorio Lo Presti, Giorgia Meineri, Biagina Chiofalo

**Affiliations:** 1 Department of Veterinary Sciences, University of Messina, 98168 Messina, Italy; marianna.oteri@unime.it (M.O.); fgresta@unime.it (F.G.); vittorio.lopresti@unime.it (V.L.P.); 2 Department of Drug Science and Technology, University of Turin, 10125 Torino, Italy; annalisa.costale@unito.it; 3 Department of Veterinary Sciences, University of Turin, 10095 Grugliasco, Italy; giorgia.meineri@unito.it

**Keywords:** amaranth, germplasm, agronomic traits, proximate composition, fatty acids, nutritional indices, phenolic compounds, antioxidant activity, feedstuff

## Abstract

With the aim to explore the use of *A. hypochondriacus* seeds for animal feeding, the agronomic traits, nutrients, and bioactive compounds of four accessions with different origin (India, Nebraska, Iowa, and Pennsylvania) grown in a Mediterranean environment were studied. Proximate composition was determined using the official methods of analyses, fatty acid profile by gas chromatography, total phenolic content (TPC) and the scavenging activity (DPPH^•^ and ABTS^•+^) by colorimetric method. A one-way ANOVA model was performed to determine the differences between accessions. The four *A. hypochondriacus* accessions showed interesting seed yield results. No significant differences were observed for crude protein and crude fiber; the oil content showed the significant highest values in the seeds from Nebraska and Pennsylvania, but their nutritional characteristics were significantly different. The accession from Nebraska showed the highest oleic and linoleic acid levels, the highest values of polyunsaturated fatty acids, the best atherogenic and thrombogenic indices and hypocholesterolemic/hypercholesterolaemic ratio, and the highest TPC content. The accession from Pennsylvania showed the highest antioxidant activity and lowest peroxidation index. On the whole, *A. hypochondriacus* seeds can be used as pseudo-cereal to balance the animal diet and the accession should be chosen according to the different metabolic pathways of unsaturated fatty acids in ruminant and monogastric animals.

## 1. Introduction

*Amaranthus* genus belongs to the *Amaranthaceae* family and is considered an invasive fast-growing species with a high capability of colonization due to the strategic adaptive seed germination [1,2]. Anyway, it is also considered a pseudo-cereal [3] and the three well-known main species are *Amaranthus hypochondriacus*, *Amaranthus cruentus*, and *Amaranthus caudatus*. They grow in temperate and tropical regions and have some agronomic advantages, namely, rapid growth, the ability to adapt to unfavorable growing conditions, and tolerance to high temperatures [4].

The recent interest for this plant genus is due to its high nutritional value of seed. In fact, *Amaranthus* is a promising plant that may provide proteins of high quality, high levels of unsaturated fatty acids, and various other valuable constituents [5]. Its seeds exhibit a valuable combination of nutritional traits, showing a higher protein and oil content compared to the conventional cereals such as corn [6] or sorghum [7] and a fatty acid profile (i.e., oleic acid) with beneficial effects for cardiovascular diseases, hypertension, hypercholesterolemia, diabetes, cancer, and inflammations, and contribute to immunity strengthening [4,8].

Furthermore, amaranth contains interesting compound deriving from the secondary metabolism, which shows health beneficial effects in human and animal diet [9]. Over the past fifteen years, some research have been carried out on the content of phenolic compounds of amaranth seeds [10] and on their functional and bioactive properties [11,12,13,14].

Seven different phenolic acids in *Amaranthus cruentus* seed have been identified: caffeic, ferulic, sinapic, *p*-coumaric, cinnamic, p-hydroxybenzoic, and vanillic acids [15]. The occurrence of ferulic acid in amaranth seeds might be considered as beneficial to health from the dietary point of view, because ferulic acid is considered a cholesterol-lowering, anti-thrombosis, anti-inflammatory, and anti-cancer factor [16]. The natural functions of hydroxycinnamic acids seem related to stabilization and protection of grain lipids [17]. The presence of caffeic, protocatechuic, and ferulic acids was also confirmed in *Amaranthus hybridus* seeds [18]. 

To our knowledge, no data are reported on the phenolic profile of *A. hypocondriacus* seed. However, information on the phenolic acid profile is essential to assess the relevance of *A. hypochondriacus* as potential source of dietary antioxidants. In fact, amaranth is becoming more and more important as animal feed and it could become a possible substitute for traditional cereals [19].

The inclusion of amaranth grain in feedstuff for many animals including cattle, chickens, pigs, and rabbits depends on its high nutritive and functional values as well as on its high digestibility and/or degradability [20]. Studies showed that the amaranth grain could be considered a valuable feed for rabbits [21], pigs [22], poultry [23], especially in the tropical and sub-tropical regions. The results on in vivo performance varied considerably within-species due to age, feed formulation, and processing method used [24,25,26,27,28,29,30]. In a previous paper [31], Gresta et al. underlined that amaranth can be considered an alternative livestock feed to oilseed and to traditional cereal grains in the Mediterranean area. Among *Amaranthus* spp., *A. hypochondriacus*, is known for its ability to tolerate stressful conditions and produce highly nutritious seeds with the highest oil and the lowest fiber levels [32]. In fact, this species possesses a considerable protein content with an optimal amino acid balance, and an outstanding profile of the crude fat [32], due to the high content of bioactive compounds such as polyunsaturated fatty acids, both of the n3- and n6-PUFA series, which reduce the incidence of cardiovascular diseases [33]. Akin-Idowu et al. [34] comparing the phenolic compounds and antioxidant activity of five grain amaranth species (*A. caudatus, A. cruentus, A. Hybrid, A. hypochondriacus* and *A. hybridus*) observed that *A. hypochondriacus* and *A. hybridus* had the highest value together with the highest DPPH activity. Barba de La Rosa et al. [35] reported that polyphenols of *A. hypochondriacus* in which sugar groups are beta-linked, are easily degraded in the intestine of humans and animals underlining their potential impact on its nutraceutical property. As far as *A. hypochondriacus* grain utilization in animal feeding is concerned, different studies have been performed [36,37,38,39] without adverse effects on performance.

With this in mind, the aim of this study is to characterize the agronomic characteristics, oil content, fatty profile, antioxidant activity, and total phenolic content of four accessions of *A. hypochondriacus* seeds with different origin (from India, Nebraska, Iowa and Pennsylvania), grown in the same area, in order to increase the knowledge of this plant as a source of nutrients and bioactive compounds (essential fatty acids and polyphenols) for animal feeding.

## 2. Materials and Methods

### 2.1. Field Experiment and Plant Material

The trial was performed in 2014 in Bovalino (RC) (20 m a.s.l. 38°08′ N, 16°10′ E) in the southern part of Calabria (Italy). Four accessions of Amaranthus hypochondriacus obtained from the USDA (Washington, DC, USA) seed bank were studied in a sandy-loam soil.

Seeds were previously grown in a nursery and then transplanted in the field. Sowing was performed manually on 21st March inside a nursery in expanded polystyrene trays and kept at 26 °C and 85% (±5%) RH. Four days later the plants with four true leaves were transplanted in the field with a plant density of 10 plants per m^2^ (1.0 × 0.10 m). In a randomized block design three time replicated with plots of 9 m^2^ (3 × 3 m) was adopted. Seed density and plot dimensions were forced by the exiguous amount of seed received from the seed bank.

The soil was shallow plowed and fertilized with 40 kg/ha of N, 80 kg/ha of P_2_O_5_, and 60 kg/ha of K_2_O before transplant. Just before anthesis, a further supply of 80 kg/ha of N as ammonium nitrate was broadcasted. Plants were irrigated with a total volume of 3200 m^3^/ha supplied with a drip system. Harvest was staggered from 5 to 11 July in relation to the degree of maturation of the different accessions, just before seed shattering. Weeds were managed by hand during the trial and seeds were finally threshed with a laboratory thresher. 

During the trial, temperatures and rainfalls were recorded by a data logger placed next to the experimental field.

The average temperature ranged from 17.7 °C at sowing to 25.2 °C at harvest. The lowest temperature was recorded at the end of April (14.6 °C), while the highest was recorded at the end of June (30.1 °C). As usually happens in spring—summer period in the Mediterranean areas, rainfall was inconsistent (26.4 mm).

### 2.2. Solvents and Chemicals

Sulfuric acid (H_2_SO_4_), 72% and 95–98%; hydrogen peroxide solution (H_2_O_2_), 30%; boric acid (H_3_BO_3_) 1% with indicator; sodium hydroxide (NaOH), 40%; anhydrous NaOH in pellets and hydrochloric acid (HCl), 0.1 N were all purchased from Carlo Erba (Carlo Erba Reagents S.r.l., Milano, Italy); catalysts Kjeltabs Cu/3,5 were obtained from the FOSS (FOSS, Padua, Italy). HCl 37%, petroleum ether (bp: boiling point 40–60 °C), methanol, and n-hexane were provided by Honeywell Fluka (Fisher Scientific Italia, Milan, Italy) and N-free filter paper (Cytiva Carta B-2 Parchment, Parchment Paper, B-2 grade) from Fisher Scientific Italia (Milan, Italy). Supelco 37 Component FAME Mix, Folin–Ciocalteu phenol reagent (FCR), gallic acid, 2,2-diphenyl-1-picrylhydrazyl (DPPH^•^), 2,2-azinobis (3-ethyl-benzothiazoline-6-sulfonic acid) (ABTS), potassium persulphate, 6-hydroxy-2,5,7,8-tetramethy-chromam-2-carboxylic acid (Trolox), and sodium carbonate were obtained from Sigma-Aldrich (Merck KGaA, Darmstadt, Germany). Solvents and chemicals were all of analytical grade.

### 2.3. Chemical Analysis

Determination of moisture and ash content in amaranth seeds was performed by using an automated thermogravimetric analyzer (TGA-701), purchased from LECO Corporation (Milano, Italy), measuring the material weight loss as a function of temperature in a controlled environment until constant weight. Moisture measurement was determined on 2 g of ground amaranth from sample weight loss after oven drying at 103 °C for 4 h [40]. Ash content determination was performed according to the official method calculating the weight remaining after heating 2 g of sample at 600 °C for 2 h [41].

Nitrogen content was determined with a Kjeltec system (Foss) using Kjeldahl procedure [41]. The method consisted in a digestion of 1 g of ground sample with concentrated sulfuric acid (95–98%) and hydrogen peroxide solution (30%), using two catalyst tablets, for 1 h at 420 °C. After cooling at room temperature, the mineralized sample was diluted with H_2_O in the automatic apparatus and neutralized using sodium hydroxide (40%). Subsequently, the solution was distilled using boric acid 1% with indicator and titrated using HCl 0.1 N. The residue of analysis obtained as Kjeldahl nitrogen content was converted to percent of crude protein using a conversion factor of 6.25 [41]. Determination of total lipid content of the four accessions of *A. hypochondriacus* seeds was carried out by using Soxtec™ 8000 Extraction system and Hydrotec™ 8000 Hydrolysis System purchased from FOSS (Padua, Italy) [42].

Lipid extraction of each accession of *A. hypochondriacus* was performed using petroleum ether (bp, 40–60 °C) for 6 h by the Soxtec system after acid hydrolysis in Hydrotec system with 3 N HCl. All extracts were dried in a ventilated oven until constant weight and the total lipid extraction yield were determined gravimetrically.

Crude fiber contents were determined according to the official method [41]. Initially, 2 g of sample was degreased three times with petroleum ether for a cold extraction, and pre-heated H_2_SO_4_ 1.25% was added until a uniform boil was attained and was let to boil for 30 min. At this point, the sample was filtered and washed with warm water for three times. Then, pre-heated NaOH 1.25% was added and let to boil for 30 min. At this point, the sample was washed with warm water and H_2_SO_4_ 1.25% and filtered. The crucible containing the sample was dried for 2 h at 130 °C and weighed. Furthermore, the sample was incinerated for 2 h at 550 °C, cooled in a desiccator and weighed. The determination of crude fiber was gravimetrically carried out.

Each accession of *A. hypochondriacus* samples was analyzed in triplicate.

### 2.4. Fatty Acids Analysis and Nutritional Indices

Fatty acid (FA) profiles evaluation, as fatty acid methyl esters (FAMEs), was performed after a direct trans-methylation treating the obtained lipid extracts with 2 mL of a sulfuric acid: methanol (1:9, *v/v*) mixture for 1 h at 100 °C [43]. After cooling to room temperature, the mixture was treated with 1 mL of n-hexane for the extraction of FAMEs. The supernatant, containing the methyl esters, was filtered using nylon filters 0.45 μm, transferred into a vial, and injected into a gas chromatographic (GC) system equipped with flame ionization detector (FID) for quali-quantitative FAMEs analysis, according to a Gresta et al. [31].

Chromatographic separations of FAMEs were carried out by means of a GC-FID (TRACE 1310) system equipped with an AI 1310 Autoinjector/AS 1310 Autosampler both from Thermo Fisher Scientific (Milan, Italy), and an Omegawax 250 (Supelco, Bellefonte, PA, USA), 30 m × 0.25 mm, (L × I.D.), 0.25 μm film thickness capillary polar column. GC conditions were set as follows: injector and detector temperature, 250 °C; injection volume, 0.5 µL; split ratio, 1:50; oven temperature program: 100 °C for 5 min, 100–240 °C at 4 °C/min in 35 min, held at 240 °C for 20 min; carrier gas (He) at a flow rate of 1 mL/min; make-up gas (N_2_) flow, 40 mL/min; H_2_ flow, 35 mL/min; air flow, 350 mL/min. Data acquisition was processed through the Chromeleon^TM^ Data System (Thermo Fisher Scientific, Milan, Italy) software (Version 7.2.9). In order to identify the individual compounds, a standard solution of 37 FAMEs (C_4_–C_24_) was analyzed under the same analytical conditions of the unknown samples; chromatogram peaks were identified by comparing the relative retention times of FAMEs in the samples with the standard solution ones. The concentrations of the single fatty acids were expressed as g/100 g, considering 100 g as the summation of the areas of all the identified FAMEs.

Nutritional indices were calculated from the identified FAs using the equations proposed by Ulbricht and Southgate [33] for atherogenicity (AI) and thrombogenicity (TI) indices, while Santos-Silva et al. equation [44] was used for the calculation of the hypocholaesterolemic and hypercholaesterolemic ratio (H/H). Indices were determinated as shown in the equations below:

AI = [C12:0 + (4 × C14:0) + C16:0]/(Ʃn6-PUFA + Ʃn3-PUFA + ƩMUFA),(1)

TI = (C14:0 + C16:0 + C18:0)/[(0.5 × ƩMUFA) + (0.5 × Ʃn6-PUFA) + (3 × Ʃn3-PUFA) + (Ʃn3-PUFA/Ʃn6-PUFA)],(2)

H/H = (C18:1n9 + C18:2n6 + C20:4n6 + C18:3n3 + C20:5n3 + C22:5n3 + C22:6n3)/ (C14:0 + C16:0) (3)

Furthermore, peroxidation index (PI), which expresses a measure of peroxidation susceptibility and peroxidative lipid damage for a particular phospholipid membrane, was calculated, taking into account the fatty acids composition and using the formula shown below [45]:

PI = (% dienoic × 1) + (% trienoic × 2) + (% tetraenoic × 3) + (% pentaenoic × 4) + (% hexaenoic × 5)(4)

### 2.5. Total Phenolic and Antioxidant Activity Analyses

Determination of total phenolic content (TPC) and antioxidant capacity was carried out on milled *A. hypochondriacus* seeds.

According to López-Mejía et al.’s method [46], 10 g of sample was treated with 150 mL of methanol and extracted in darkness for 15 h at room temperature. The sample, once extracted, was filtered and washed, three times, with methanol; at last, the solvent was removed using a rotary evaporator. Extraction yields (%) were calculated gravimetrically considering seeds weight and dry extracts.

Total phenolic content (TPC) was determinate by the Folin–Ciocalteu colorimetric method [10] as follows: 250 μL of methanolic solution of extract (5 mg/mL), 250 μL of FCR, 500 μL of 10% sodium carbonate solution, and 4 mL of water, were mixed. The mixture was incubated for 25 min in a dark environment at room temperature, centrifuged 5 min at 5000 rpm, and the absorbance was measured at a wavelength of 725 nm. The results were expressed as mg of gallic acid equivalents (GAE, mg/g) per g of sample. Each determination was performed in triplicate. Methanol extracts of polyphenols were analyzed for their antioxidant capacity by DPPH^•^ and ABTS^•+^ assays. The DPPH^•^ scavenging activity was evaluated according to the procedure described by Brand-Williams et al. [47]. The DPPH^•^ radical is stable in methanol solution; extracts of antioxidants scavenge the DPPH^•^, and the reduction of DPPH^•^ is monitored by the decrease of the absorbance at wavelength 517 nm. An aliquot of methanol solution (0.1 mL) containing different concentrations (from 2–10 mg/mL of initial sample), was added to 0.25 mL of 1 mM DPPH and 2 mL of methanol, mixed and the absorbance was measured after 20 min (λ=517 nm) by a Cary 60 UV-Vis spectrophotometer (Agilent Technologies, Santa Clara, CA, USA). Data were used to determine the quantity (μg) of polyphenols needed to scavenge 50% of the DPPH^•^ (EC50). Then, EC50 values, considered as dried extract concentration (mg/mL solution) needed to scavenge the 50% of initial DPPH^•^, were evaluated. The Trolox equivalent antioxidant capacity (TEAC) was determined according to the assay reported by Karamać et al. [10]. Total of 20 μL of methanolic solution of extracts (20 mg/mL) or methanolic Trolox standard solution was mixed with 2 mL ABTS^•+^ diammonium salt radical cation, vortexed and heated at 30 °C. The absorbance was read at λ = 734 nm by a Cary 60 UV-Vis spectrophotometer (Agilent Technologies, Santa Clara, CA, USA) within 6 min. The final results were expressed as μmol TE (Trolox equivalents)/g of seed.

### 2.6. Statistical Evaluation

A one-way ANOVA model was adopted to analyze the data. When significative difference was detected among accessions, Tukey’s (HSD) test was applied, using DSAASTAT v. 1.1 software [48]. To ensure normality, percentage values were previously arcsin square root transformed; in tables, percentage data were reported.

## 3. Results

The four accessions of *A. hypochondriacus* from India, Nebraska, Iowa, and Pennsylvania are listed in Table 1 with plant inventory code.

### 3.1. Productive Traits

Accessions from Nebraska and Pennsylvania showed the highest seed weight per plant (43.5 g, on average) significantly higher than accessions from India and Iowa (27.7 g, on average) (Table 1). On the other hand seed weight was higher in the accessions from India and Iowa (0.85 g for 1000 seeds, on average), even though not significatively different from Nebraska (0.77 g).

### 3.2. Chemical Composition

Table 2 shows the mean values of the chemical composition in the four studied accessions of amaranth seed. Crude protein and crude fiber showed a similar (*p* > 0.05) content among all the *A. hypochondriacus* accessions. Significant differences between Amaranth accessions were also observed for the oil content and ash (Table 2). Nebraska (USA) and Pennsylvania (USA) showed a significantly higher (*p* < 0.05) oil content compared to India. The ash content was significantly (*p* < 0.05) higher in the accessions from Nebraska (USA) and Iowa (USA) than that from Pennsylvania (USA).

### 3.3. Fatty Acids, Total Phenolic Contents, and Antioxidant Activity

Fatty acid composition in the four amaranth seeds is presented in Table 3. Linoleic (C18:2n6), palmitic (C16:0), and oleic (C18:1n9) acids were the dominant fatty acids in all samples. The linoleic acid (C18:2n6) exhibited the significantly (*p* < 0.05) highest level in the accessions from India PI 558499, Nebraska, and Iowa. The palmitic (C16:0) showed a significantly (*p* < 0.05) higher value in the accession from Pennsylvania than those from Nebraska, Iowa, and India. The oleic acid (C18:1n9) showed a significantly (*p* < 0.05) higher value in the accession from Nebraska than those of Pennsylvania and India, while accession from Iowa showed a similar content among the four accessions. Pennsylvania showed the significantly (*p* < 0.05) highest value of stearic acid (C18:0). The myristic (C14:0), palmitoleic (C16:1), heptadecanoic (C17:0), cis-11-octadecenoic acid (C18:1n7) an isomer of oleic acid, the alpha-linolenic (C18:3n3), arachic (C20:0), and docosanoic (C22:0) acids were lower than 2% in all amaranth accessions (Table 2). 

Table 4 covers the fatty acid classes, the fatty acid ratios, and the quality indices, AI and TI. The accession from Nebraska showed the significantly (*p* < 0.05) lowest value of SFAs; MUFAs did not show any statistical differences (*p* > 0.05) among the studied accessions, while the accession from Pennsylvania showed the significantly (*p* < 0.05) lowest value of PUFAs and, in particular, n6-PUFAs, and the significantly (*p* < 0.05) highest value of SFAs (Table 4). Consequently, the SFA/UFA ratio showed the significantly (*p* < 0.05) highest value in the accession from Pennsylvania and the lowest in that from Nebraska (Table 4). As regards the nutritional indices, strictly related to the fatty acid profile, the AI, TI, and H/H showed the significantly (*p* < 0.05) best values in the accession from Nebraska and the worst values in that from Pennsylvania, and the PI, showed the significantly (*p* < 0.05) lowest value in the accession from Pennsylvania (Table 4).

Table 5 showed the total phenolic content (TPC) and the antioxidant power (DPPH^•^ and ABTS^•+^) in the four different accessions of *A. hypochondriacus.* The accession from Nebraska showed the significantly (*p* < 0.05) highest TPC content whereas, the accession from Iowa showed the significantly (*p* < 0.05) lowest value.

As regards the antioxidant power, the DPPH^•^ values showed the lowest (*p* < 0.05) level in the accession from Pennsylvania and the highest (*p* < 0.05) value in that from Iowa. Intermediate significantly different (*p* < 0.05) values were observed for the accessions from Nebraska and India. The DPPH^•^, in the different accessions of *A. hypochondriacus* varied significantly (*p* < 0.05) in this order: Iowa > Nebraska > India > Pennsylvania (Table 5). Accession from Iowa showed a high DPPH^•^ value (low antioxidant activity) in relation to the high content of PUFAs which are unstable to oxidation (Table 4). The accession from Pennsylvania, maintains a low DPPH^•^ value (high antioxidant activity) in relation to the high content of SFAs which are stable to oxidation (Table 4). From a health point of view, the accession from Nebraska represents the best accession showing the highest content of polyphenols, an intermediate DPPH^•^ value, the lowest SFA, and the highest PUFA content (Table 4); in this case, the observed TPC value can counteract the oxidation of the PUFA present in the plant. The ABTS^•+^ radical-scavenging activity showed a significantly (*p* < 0.05) higher value (high antioxidant activity) in the accession from Pennsylvania than those determined in the other accession which ranged from 1.75 to 2.22 μmol TE /g seeds, according to the highest SFA content in this accession, as stated above for the DPPH^•^ value.

## 4. Discussion

Interesting seed yield was recorded in our trial. Compared to the literature data, in our trial seed yield was higher than that reported on the same species by El Gendy et al. [5] and by Pulvento et al. [49]. Our data resulted in agreement to the result obtained by Gimplinger et al. [42] with the same plant density. The 1000 seed weight is also in agreement with Gimplinger et al. who found a 1000 seed weight ranging from 0.55 to 1.04 g [50]. Seed yield seems to be inversely related to the 1000 seed weight. In fact, the accessions with the highest seed weight per plant showed a lower 1000 seed weight.

The crude protein content in the four studied accessions of *A. hypochondriacus* seeds (17 to 18 g/100 DM) is in agreement with the value reported by Rutkowska [51] (17.9% DM) and higher than the mean value (15.6% DM, on average) observed by Barba de la Rosa et al. [35] in four varieties of *A. hypochondriacus* grown in Mexico. The crude protein content is also within the range reported in literature for amaranth seeds, which ranges from 13 to 18% [46,52,53,54,55,56]. Moreover, as a general rule, we could affirm that *A. hypochondriacus* exhibits a higher content of crude protein when compared to cereals such as corn (10.3% of DM), wheat (14% DM), and rice (8.5% DM) [54]. 

In the present study, the oil content ranges from 5 to 7% in the four different accessions of *A. hypochondriacus* according to data reported by Grobelnik Mlakar et al. [57] (6.1–7.3%) in five observations of *A. hypochondriacus* and by Petkova et al. [58] in amaranth seeds with different origin, Indian (6.9% DM) and Turkish (7.4% DM). Barba de la Rosa et al. [35] and Rutkowska [51] found a lipid content within the range of 7.7–8.9%, in different accession of *A. hypochondriacus*. Our data resulted also in agreement with the data found by Gresta et al. [31] in eight accessions of *A. cruentus* seeds (5.6–7.3%, as fed). On the whole, as a general observation, *A. hypochondriacus* exhibits a higher oil content (5.7% DM, on average) when compared to cereals such as corn (4.5% DM), wheat (2.1% DM), and rice (2.1% DM) [54].

Ash content (3.3–3.5%, mean value 3.4% DM) in the four *A. hypochondriacus* accessions is within the range recorded by Grobelnik Mlakar et al. [57] (3.3–3.4%), but lower than the mean value (4.1% DM) observed by Rutkowska [51]. Our values are also in agreement with those reported by Ogrodowska et al. in *A. cruentus* seeds [52]. Compared to other cereals, the ash content exhibits a higher content (3.42% DM, on average) than corn (1.4% DM), wheat (1.9% DM), and rice (1.4% DM) [54].

Our results of crude fiber content (4.8–5.9%) agree with the results obtained by Gobelin Mlakar et al. (4.9%) [57] and are higher than that reported by Orona-Tamayo and Paredes-López (2.2%) [54]. Moreover, comparing the crude fiber content with that obtained in other *Amaranthus* spp., our data resulted lower than that reported by Pedersen et al. in *A. caudatus* grain (8.0%) [55]. Finally, the crude fiber exhibits a higher content (5.47% DM, on average) in comparison to cereals such as corn (2.3% DM), wheat (2.6% DM), and rice (0.9% DM) [54]. 

In relation to the chemical composition of amaranth seeds, different studies were carried out on animal nutrition. Jalč et al. [36] in a feeding trial on sheep have demonstrated that *A. hypochondriacus* grain can represent a partial substitute of barley. Kubelková et al. [59] observed an increase of the microbial protein production in the effluents of ruminants fed with *A. hypochondriacus* grains. Moreover, amaranth can represent a valuable ingredient in the conventional rabbit feeding [21,60,61]. Reddy and Reddy [62] reported a favorable influence on growth in rabbits fed a ration containing *A. hypochondriacus* grain. Bamikole et al. [63] reported that unthreshed mature grain amaranth could be included in rabbit feeding up to a level of 10%. Similar results were also obtained with other *Amaranth* spp.; Molina et al. [21] suggested that *A. dubius* could be used as a fiber and protein source for rabbit diets in subtropical and tropical regions. Moreover, Molina et al. [64] in rabbits fed with an increasing level of *A. dubius,* observed an increase in meat protein and fat contents. It has been also demonstrated that amaranth seeds, which have higher amount of protein than cereal grains and similar energy content, can be used as raw material in the finisher diets of broilers [65]. Waldroup et al. [38] showed that *A. hypochondriacus*, more than the *A. cruentus* grain, may be consumed by broiler chickens in substitution of the traditional control diet, based on maize-soybean meal. Kabuage et al. [23] observed that a pelleting of the amaranth in the diets of 8-week-old broiler chicks resulted in an increase of the body weight, feed intake, feed efficiency, and carcass fat.

Some unsaturated fatty acids, such as linoleic and linolenic acid, are exogenous acids essential for the animal body, playing an important role in regulating the immune-inflammatory system, hormone synthesis, cell membrane structure, and regulation of their permeability [66]. According to the literature [67], *A. hypochondriacus* exhibited a higher level of essential fatty acids, linoleic acid, and alpha-linolenic content than those reported by Gresta et al. [31] in eight accessions of *A. cruentus* (26.8% and 0.37%, respectively). Consequently, also the total polyunsaturated fatty acids, both n6- and n3-PUFA, were higher in the *A. hypochondriacus* than those recorded by Gresta et al. [31] (27.19%, 26.82%, and 0.37%, respectively). 

Overall, our results suggest the possibility of replacing conventional cereals in pig feeding with the amaranth grains, owing to their relatively high dietary protein content and good dietary fiber content [68] and to their favorable lipid composition, in particular essential fatty acids which can be effective in the production of healthy pork as they modify the fatty acid composition of animal tissues [69].

As regards the nutritional aspects, in comparison with the main cereals used in animal feeding, the fatty acid composition of *A. hypochondriacus* showed a lower content of linoleic acid (omega 6) and linolenic acid (omega 3), and a higher content of oleic acid (omega 9) in comparison with maize (*Zea mays*) [70], wheat (*Triticum aestivum* and *Triticum durum*), barley (*Hordeum vulgare*), rye (*Secale cereale*), and triticale (*Triticosecale*) [71]. It must be mentioned that oleic acid has a beneficial effect on heart-diseases reducing the oxidation of LDL cholesterol, slowing the progression of atherosclerosis, and limiting cardiovascular risk [72,73]. The potential use of amaranth seeds in medicine to alleviate atherosclerotic disorders is well-known [32]. Our data evidenced a higher content of UFAs (63.13% on average) than the SFAs (35%) even if the SFA/UFA ratio appears significantly higher (0.56, on average) than that observed in literature on *A. hypochondriacus* grains by El Gendy et al. [5] (0.31), He et al. [67] (from 0.26 to 0.27), He et al. [74] (0.37), and Jahaniaval et al. [75] (from 0.37 to 0.40). Nevertheless, our data on the SFA/UFA ratio in the four studied accessions of *A. hypochondriacus* seeds appeared lower than that recorded by Gresta et al. [31] in *A. cruentus* grown in a Mediterranean environment (0.61, on average). Concerning the nutritional indices, there are no references in literature of the AI, TI, and H/H indices in the oil from amaranth seeds except for the values reported by Gresta et al. [31] in eight accessions of *A. cruentus* which showed higher and, therefore, worse values than those recorded in our trial on *A. hypochondriacus* seeds. The atherogenic (AI) and thrombogenic (TI) indices applied by Ulbright and Southgate [33] indicate the relationship between the sum of three SFAs, lauric, myristic, and palmitic acids for AI and myristic, palmitic, and stearic acids for TI, and the sum of the main classes of unsaturated fatty acids, which are antiatherogenic. Therefore, AI is a measurement of the level of the atherogenicity while TI measures the level of thrombogenicity. A further beneficial effect of *Amaranthus* spp. on health can be ascribed to its hypocholesterolaemic action [8]. To evaluate the oil nutritional value, a further approach is represented by the ratio of the hypocholesterolaemic/hypercholesterolaemic fatty acids (HH), calculated on the basis of the functional effects of individual fatty acids on the cholesterol metabolism [76]. Our data showed significant differences for AI, TI, and H/H ratio among the accessions, resulting in the best values (0.43, 0.91, 2.47, respectively) in the accession from Nebraska for all the nutritional indices. Although *A. hypochondriacus* showed a low amount of n-3 PUFAs, the nutritional indices testify that the consumption of amaranth oil leads to improved cardiovascular health, decreases in bad cholesterol from blood, and helps to prevent metabolic imbalance [77]. Kabiri et al. [78,79] observed a significant reduction of the atherogenic index in atherosclerotic rabbits fed with amaranth extract supplementation. Plate and Arêas [80] demonstrated that the cholesterol-lowering effect of *Amaranthus* reduces low-density lipoprotein and total cholesterol levels in rabbit blood. Furthermore, Punita and Chaturvedi [26] fed laying hens with *A. paniculatus* grain and red palm oil, known to be a hypercholesteremic agent, and found an increase in the linoleic acid and a reduction in the cholesterol content in the eggs of hens fed red palm oil + amaranth and raw amaranth groups. Króliczewska et al. [81] studied the influence of 0, 2, 5, and 10% supplementation of *A. cruentus* grain on the biochemical and hematological blood parameter values of laying hens. The increase in inclusion level of amaranth determined a decrease in the low-density lipoprotein cholesterol.

In the four accessions of *A. hypochondriacus* seeds studied, the peroxidation index (PI) was significantly influenced by the accessions and showed an increase in relation to the increase in PUFA content, as observed by Gresta et al. [31] in *A. cruentus* grown in a Mediterranean environment. The PI average value reflected the different PUFA contents, being higher in our samples (34.4, on average) than that calculated by Gresta et al. [31] in *A. cruentus* seeds (27.6, on average). The peroxidation index (PI), that represents the relative rate of peroxidation reaction, showed the lowest content in accession from Pennsylvania. 

Total polyphenol content in the four studied varieties of amaranth seed is in agreement with the data recorded by Akin-Idowu et al. [34] in five species of *Amaranthus (A. caudatus, A. cruentus, A. hybrid, A. hypochondriacus* and *A. hybridus)* and, specifically, with TPC content obtained on *A. hypochondriacus* (0.30 mg GAE/g) while it is higher than that observed by Gresta et al. [31] in eight accessions of *A. cruentus* (0.26. mg GAE/g, on average). When compared with other species, our values are much lower than those obtained in other pseudo-cereals like buckwheat (3.23 mg GAE/g) and quinoa (0.72 mg GAE/g), lower than those of corn (0.49 mg GAE/g) and sesame (0.42 mg GAE/g) and similar to those obtained in soybean (0.37 mg GAE/g), oatmeal (0.35 mg GAE/g), and linseed (0.31 mg GAE/g) [82], while they are higher than that recorded in oat (0.20 mg GAE /g) [83].

As regards, the scavenging activity (DPPH^•^ and ABTS^•+^), the comparison with the results reported by literature must be very cautious since there are many assays for total antioxidant determination and each of them has its limitations [84]. In this study, we applied the capacity to scavenge the “stable” free radical 2,2 diphenyl-1-picrylhydrazyl (DPPH^•^) [47] and 2,2′-Azino-bis(3-ethylbenzothiazoline-6-sulfonic acid) diammonium salt radical cation (ABTS^•+^) frequently used to estimate the total antioxidant capacity of natural products, including crude extracts, polyphenols, phenolic acids, flavonoids, and others [85]. Results of the two assays confirmed the best antioxidant activity for the accession of Pennsylvania.

Comparing DPPH^•^ to the data reported by Gresta et al. [31] in *A. cruentus* cultivated in the same environment where our trial was carried out, the range appears quite similar (0.30–0.50 mg/mL). Conversely, our DPPH^•^ mean value is higher and that of ABTS^•+^ is lower than those reported by Karamać et al. [10] (DPPH^•^: 0.21 mg/mL; ABTS^•+^: 7.62 μmol TE /g), testifying a lower antiradical activity of *A. hypochondriacus* than that of *A. caudatus* seeds. Nevertheless, Peiretti demonstrated that amaranth seeds in the diet of rabbits could be used as an effective natural antioxidant supplement capable of protecting cellular membranes against oxidative damage [30]. Moreover, Longato et al. [86] observed a significant higher serum antioxidant power and a lower serum lipid peroxidation levels in broilers fed with diets containing 5 and 10% of amaranth grain than those receiving a diet without supplementation.

## 5. Conclusions

The productive traits of the four *A. hypochondriacus* accessions show valuable results that suggest that this species could be considered as a possible alternative crop to the traditional cereals for irrigated Mediterranean environment.

The chemical composition of the four accessions of *A. hypochondriacus* seed showed that this pseudo-cereal can be used to balance the animal diet. Protein and fat showed a higher content than the most important cereals. Moreover, not only the quantity, but also the lipid composition of *A. hypochondriacus* grains is appreciable for its nutritional value and its beneficial health properties. *A. hypochondriacus* seeds showed a high content of unsaturated fatty acid of the n6 (linoleic acid) and n9 (oleic acid) series, therefore, this seed oil can be used in animal nutrition to enrich the products of animal origin with fatty acids. Furthermore, *A. hypochondriacus* seed showed a higher total phenolic content than oat and a comparable content with soybean, oatmeal, and linseed, revealing that the use of amaranth grain can be considered as an effective natural antioxidant supplement in animal nutrition that helps protect cell and tissues from damaging effects of free radicals and oxidative stress.

Among amaranth accessions, Nebraska and Pennsylvania showed the highest oil content and emerged as the best healthy ingredient for animal feeding. In particular, the accession from Nebraska showed the highest levels of oleic and linoleic acids, the highest content of polyunsaturated fatty acids, the best atherogenic, thrombogenic indices, and hypocholesterolaemic/hypercholesterolaemic ratio, and the highest TPC content. The accession from Pennsylvania, showed the lowest peroxidation index and DPPH^•^ value and, therefore, the highest antioxidant activity.

In this light, we can affirm that the choice of *A. hypochondriacus* seed as a substitute for traditional cereals, due to its potential source of health- promoting bioactive compounds, should be encouraged and different accessions could be chosen according to the different metabolic pathways of ruminants and monogastric animals.

## Figures and Tables

**Table 1:** Four studied *Amaranthus hypochondriacus* accessions.

Accession	Origin	Seed Weight per Plant (g)	1000 Seed Weight (g)
PI 477915	India	28.0 ^b^	0.85 ^a^
PI 558499	USA, Nebraska	43.3 ^a^	0.77 ^ab^
PI 568125	USA, Iowa	27.4 ^b^	0.84 ^a^
PI 572256	USA, Pennsylvania	43.7 ^a^	0.68 ^b^

PI = plant inventory.

**Table 2:** Mean values of three replications of the chemical composition (g/100 on dry matter) in the four accessions of *A. hypochondriacus* seed.

Accession	Origin	Moisture	Crude Protein	Oil	Crude Fiber	Ash
PI 477915	India	10.40	18.30	5.39 ^b^	4.84	3.36 ^ab^
PI 558499	USA, Nebraska	10.20	17.60	7.23 ^a^	5.42	3.53 ^a^
PI 568125	USA, Iowa	10.30	17.30	6.00 ^ab^	5.85	3.54 ^a^
PI 572256	USA, Pennsylvania	10.20	17.70	6.87 ^a^	5.77	3.26 ^b^
Average		10.3	17.73	6.37	5.47	3.42

PI = plant inventory. Mean values with different letters (a–c) within the same column differ significantly (*p* < 0.05).

**Table 3:** Mean values of three replications of the fatty acid profile (g/100 g) * in the four accessions of *A. hypochondriacus* seed.

Accession	Origin	C14:0	C16:0	C16:1	C17:0	C18:0	C18:1n9	C18:1n7	C18:2n6	C18:3n3	C20:0	C22:0	Other
PI 477915	India	0.75 ^b^	27.00 ^b^	0.23	0.41 ^b^	5.89 ^b^	26.40 ^b^	1.42	34.00 ^a^	0.62 ^a^	0.85 ^b^	0.42 ^b^	1.96 ^b^
PI 558499	USA, Nebraska	0.67 ^c^	25.30 ^c^	0.25	0.41 ^b^	5.03 ^d^	29.20 ^a^	1.41	34.30 ^a^	0.50 ^bc^	0.75 ^b^	0.36 ^c^	1.77 ^bc^
PI 568125	USA, Iowa	0.59 ^d^	26.20 ^bc^	0.29	0.36 ^c^	5.38 ^c^	28.40 ^ab^	1.43	33.80 ^a^	0.54 ^b^	0.82 ^b^	0.40 ^b^	1.70 ^c^
PI 572256	USA, Pennsylvania	0.83 ^a^	29.00 ^a^	0.44	0.51 ^a^	6.39 ^a^	26.60 ^b^	1.43	30.70 ^b^	0.44 ^c^	1.03 ^a^	0.49 ^a^	2.20 ^a^
Average		0.71	26.88	0.30	0.42	5.67	27.65	1.42	33.20	0.53	0.86	0.42	1.91

PI = plant inventory. Mean values with different letters (a, b, c, d, ab, bc) within the same column differ significantly (*p* < 0.05). * The concentration of individual fatty acids was expressed per total fatty acid methyl esters identified. C14:0 = myristic acid; C16:0 = palmitic acid; C16:1 = palmitoleic acid; C17:0 = heptadecanoic acid; C18:0 = stearic acid; C18:1n9 = oleic acid; C18:1n7 = cis-vaccenic acid; C18:2n6 = linoleic acid; C18:3n3 = α-linolenic acid; C20:0 = arachidic acid; C22:0 = behenic acid.

**Table 4:** Mean values of three replications of fatty acid classes (g/100 g) *, ratios and quality indices in the four accessions of *A. hypochondriacus* seed.

Accession	Origin	SFA	MUFA	PUFA	SFA/UFA	n3	n6	AI	TI	H/H	PI
PI 477915	India	35.40 ^b^	28.10	34.60 ^a^	0.56 ^b^	0.62 ^a^	34.00 ^a^	0.48 ^b^	1.02 ^b^	2.20 ^b^	35.20 ^a^
PI 558499	USA, Nebraska	32.50 ^c^	30.90	34.80 ^a^	0.50 ^c^	0.50 ^bc^	34.30 ^a^	0.43 ^c^	0.91 ^c^	2.47 ^a^	35.30 ^a^
PI 568125	USA, Iowa	33.80 ^bc^	30.10	34.40 ^a^	0.52 ^bc^	0.54 ^b^	33.80 ^a^	0.44 ^bc^	0.96 ^bc^	2.34 ^ab^	34.90 ^a^
PI 572256	USA, Pennsylvania	38.20 ^a^	28.50	31.10 ^b^	0.64 ^a^	0.44 ^c^	30.70 ^b^	0.54 ^a^	1.17 ^a^	1.94 ^c^	31.60 ^b^
Average		35.00	29.40	33.73	0.56	0.53	33.20	0.47	1.02	2.24	34.25

PI = plant inventory. Mean values with different letters (a, b, c, ab, bc) within the same column differ significantly (*p* < 0.05). * The concentration of fatty acid classes was expressed per total fatty acid methyl esters identified. SFA = saturated fatty acids; MUFA = monounsaturated fatty acids; PUFA = polyunsaturated fatty acids; n3 = n3-polyunsaturated fatty acids; n6 = n6-polyunsaturated fatty acids; SFA/UFA = saturated/unsaturated fatty acid ratio; AI = atherogenic index; TI = thrombogenic index; H/H = hypo-/hypercholesterolaemic ratio; PI = peroxidation index.

**Table 5:** Mean values of three replications of antioxidant properties in the four accessions of *A. hypochondriacus* seed.

Accession	Origin	TPC	DPPH^•^	ABTS^•+^
PI 477915	India	0.31 ^b^	0.42 ^c^	1.84 ^b^
PI 558499	USA, Nebraska	0.40 ^a^	0.45 ^b^	2.22 ^b^
PI 568125	USA, Iowa	0.24 ^c^	0.54 ^a^	1.75 ^b^
PI 572256	USA, Pennsylvania	0.30 ^b^	0.34 ^d^	3.08 ^a^
Average		0.31	0.44	2.22

PI = plant inventory. Mean values with different letters (a, b, c, d) within the same column differ significantly (*p* < 0.05). TPC = total phenolic content expressed as gallic acid equivalents (GAE mg/g seeds); DPPH^•^ scavenging activity expressed as E C50 (concentration of dried extract mg/mL solution); ABTS^•+^ scavenging activity expressed as μmol TE (Trolox equivalents)/g seeds).

## Data Availability

Not applicable.
